# Costs and Cost Effectiveness of Three Approaches for Cervical Cancer Screening among HIV-Positive Women in Johannesburg, South Africa

**DOI:** 10.1371/journal.pone.0141969

**Published:** 2015-11-16

**Authors:** Naomi Lince-Deroche, Jane Phiri, Pam Michelow, Jennifer S. Smith, Cindy Firnhaber

**Affiliations:** 1 Health Economics and Epidemiology Research Office, Department of Internal Medicine, School of Clinical Medicine, Faculty of Health Sciences, University of the Witwatersrand, Johannesburg, Gauteng, South Africa; 2 Cytology Unit, National Health Laboratory Service and Department of Anatomical Pathology, Faculty of Health Sciences, University of the Witwatersrand, Johannesburg, Gauteng, South Africa; 3 Department of Epidemiology, Gillings School of Public Health, University of North Carolina, Chapel Hill, NC, United States of America; 4 Lineberger Comprehensive Cancer Center, University of North Carolina, Chapel Hill, NC, United States of America; 5 Right to Care, Johannesburg, Gauteng, South Africa; 6 Clinical HIV Research Unit, Department of Internal Medicine, Faculty of Health Sciences, University of Witwatersrand, Johannesburg, Gauteng, South Africa; Penn State University School of Medicine, UNITED STATES

## Abstract

**Background:**

South Africa has high rates of HIV and HPV and high incidence and mortality from cervical cancer. However, cervical cancer is largely preventable when early screening and treatment are available. We estimate the costs and cost-effectiveness of conventional cytology (Pap), visual inspection with acetic acid (VIA) and HPV DNA testing for detecting cases of CIN2+ among HIV-infected women currently taking antiretroviral treatment at a public HIV clinic in Johannesburg, South Africa.

**Methods:**

Method effectiveness was derived from a validation study completed at the clinic. Costs were estimated from the provider perspective using micro-costing between June 2013-April 2014. Capital costs were annualized using a discount rate of 3%. Two different service volume scenarios were considered. Threshold analysis was used to explore the potential for reducing the cost of HPV DNA testing.

**Results:**

VIA was least costly in both scenarios. In the higher volume scenario, the average cost per procedure was US$ 3.67 for VIA, US$ 8.17 for Pap and US$ 54.34 for HPV DNA. Colposcopic biopsies cost on average US$ 67.71 per procedure. VIA was least sensitive but most cost-effective at US$ 17.05 per true CIN2+ case detected. The cost per case detected for Pap testing was US$ 130.63 using a conventional definition for positive results and US$ 187.52 using a more conservative definition. HPV DNA testing was US$ 320.09 per case detected. Colposcopic biopsy costs largely drove the total and per case costs. A 71% reduction in HPV DNA screening costs would make it competitive with the conservative Pap definition.

**Conclusions:**

Women need access to services which meet their needs and address the burden of cervical dysplasia and cancer in this region. Although most cost-effective, VIA may require more frequent screening due to low sensitivity, an important consideration for an HIV-positive population with increased risk for disease progression.

## Introduction

Cervical cancer is the most common cancer affecting women in Sub-Saharan Africa [1] and the leading cause of cancer related death among females in the region [2]. Globally, cervical cancer is the fourth most common cancer affecting women and accounts for 12% of all cancers among women in resource-limited settings [1].

Approximately 90% of all cervical cancer cases are caused by persistent infection with high-risk (oncogenic) type human papillomavirus (HPV) [3,4]. Most HPV infections resolve spontaneously within 1–2 years [5]. A study conducted in Brazil found that of women positive for HPV at enrollment, just 35% were still positive 12 months later [6]. However, HIV infection is a major risk factor for persistent HPV infection and abnormal cytological findings [7–10]. The prevalence of atypical squamous cells of undetermined significance (ASCUS) and squamous intraepithelial lesions (SIL) has been found to be 1.8 and 3.9 times greater respectively in HIV-positive women than in HIV-negative women [8].

South Africa’s HIV and HPV infection rates are among the highest globally. Adult HIV prevalence is 17.9% [11], and HPV prevalence ranges from 20.4% among women with normal cytology to 83% in women with high-grade SIL [12]. The age-standardized rates of cervical cancer incidence and mortality for the general population are 31.7 and 18.0 per 100,000 respectively [1]. However, for HIV-positive women, invasive cervical cancer rates are much higher. One treatment center in Gauteng Province documented a cervical cancer rate of 168 per 100,000 for women taking antiretroviral (ARV) therapy [13].

Early screening for cervical cancer allows for diagnosis of abnormalities at a pre-invasive and treatable stage. Screening interventions currently available globally include conventional and liquid based cytology (or a Papanicolaou (Pap) smear), visual inspection with acetic acid (VIA), and HPV testing. Cytology and HPV testing require collection of a sample of cells and “reading” or testing of the sample in a laboratory [14]. In resource limited settings, results are usually only available days or weeks after the sample collection. In contrast, VIA, which involves the application of dilute acetic acid to the cervix to visually identify lesions, can result in an immediate diagnosis (i.e. during the procedure) [15].

Use of screening approaches varies globally and is often based on resource availability and perceived cost effectiveness. In South Africa, according to national guidelines, all women should have access to free cervical cancer screenings with conventional cytology in the public sector at ten year intervals from age 30 to 50 [16]; HIV-positive women are allowed screening upon diagnosis and then annually or three-yearly depending on the results of the initial and subsequent screens [17].

Most existing studies of the cost-effectiveness of cervical cancer screening rely on models that draw input parameters from multiple and disparate sources [18–20]. Firnhaber et al. recently validated the sensitivity and specificity of conventional cytology, VIA, and HPV DNA testing for detection of cervical intra-epithelial lesion grade two or higher (CIN 2+) among HIV-positive women taking ARV treatment at an HIV clinic in Johannesburg, South Africa [13]. We collected data from the study site to estimate costs and evaluate the cost-effectiveness of the screening methods used by Firnhaber et al for detecting cases of CIN2+ among the cohort of HIV-infected women on treatment.

## Materials and Methods

### Effectiveness

**Fig 1:**
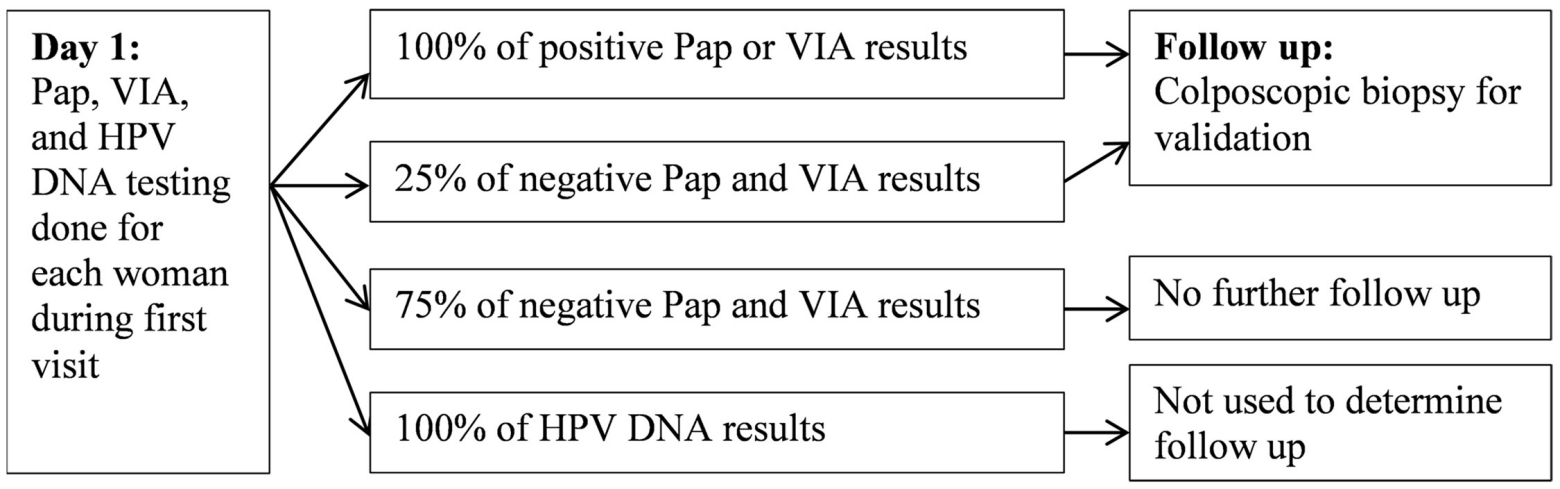
Validation study design for detection of CIN in HIV-positive women. In the validation study, which preceded the study presented here, all women were screened with all methods. After screening and diagnosis all women were followed up using study guidelines and local standards of care.

Effectiveness was defined as detected cases of CIN2+. Sensitivity and specificity for detection of this outcome by each screening method were derived from the validation study [13]. The study was carried out at an HIV treatment clinic within a large, tertiary, public hospital in Johannesburg, South Africa from November 2009 to August 2011. HIV-infected women aged 18–65 were screened using the three alternative procedures: conventional cytology (or Pap), VIA, and HPV DNA testing with Hybrid Capture (Qiagen). All women gave written consent to participate in the validation study and were screened with all three methods. Data used for the cost evaluation were de-identified. A positive Pap result or positive VIA was followed by colposcopic biopsy to allow for histological confirmation of the initial result by an anatomical pathologist (Fig 1). Approximately 25% of the women with negative Pap smears and negative VIA results also received colposcopic biopsy. HPV DNA results were received later and were not used to determine whether colposcopic biopsy would be done.

Positive Pap results were defined in two ways in the validation study. The first definition (Pap 1) followed current practice in the public sector. Positive results included: high grade SIL (HSIL), “atypical squamous cells cannot rule out high grade lesion” (ASC-H), and squamous cell carcinoma (SCC). Currently, women who are deemed positive by this definition are referred for further diagnostics and/or treatment. To address the potential for increased risk of progression to disease among HIV-positive women infected with HPV, a second definition (Pap 2) was created for the validation study. With this definition a positive result was deemed to include any non-negative result. Note that the procedure (and the associated cost) used for the Pap test was the same for Pap 1 and Pap 2. The difference was in the interpretation of the results only.

### Cost

Costs were estimated from a provider perspective. Data collection occurred between June 2013 and April 2014 at the same health facility that implemented the validation study. Staff time, supplies, and equipment required per procedure were collected through discussions with the clinic staff. At least three staff members provided input per procedure type. An average time per procedure and average supply and equipment usage were then calculated using the three responses unless there were large discrepancies in the reported information. For those cases, staff were approached a second time to discuss the discrepancies and reach a consensus.

Unit costs for supplies and equipment were obtained from facility expenditure records or other publicly available sources [21,22]. Personnel costs were drawn from public sector salary scales [23]. National Health Laboratory Service (NHLS) service charges for 2012–2013 were used to estimate laboratory costs, which included specimen transport costs and some consumables including the glass slides, fixative and lab forms. Following generally recommended methods [24], capital costs were annualized to obtain the equivalent annual cost using a discount rate of 3% and depreciation periods recommended for various categories of equipment by the South Africa Revenue Service [25]. All costs were collected in South African Rand (R), inflated to 2013 prices (where necessary) using the South African Consumer Price Index [26], and are reported here in 2013 US dollars using an average exchange rate for 2013 of 9.65 Rands per dollar [27].

Fixed costs such as infrastructure and utilities did not vary among the three screening methods and are not included in the analysis. All three screening services were offered by professional nurses who had received study-related training. The cost of this training is also not included. Professional nurses learn to collect Pap smears as part of their four-year nursing qualification in South Africa, so the cost of a professional nurse was used for collection of the Pap smear. As collection of HPV DNA samples is very similar to collection of a Pap smear, the cost of a professional nurse was also used for that service. VIA is not covered in professional nurses’ education and would require extra training, thus for this analysis the cost of a nurse with an additional two years of education, called a “primary health care (PHC) nurse,” was used for the VIA service.

### Analysis

**Fig 2:**
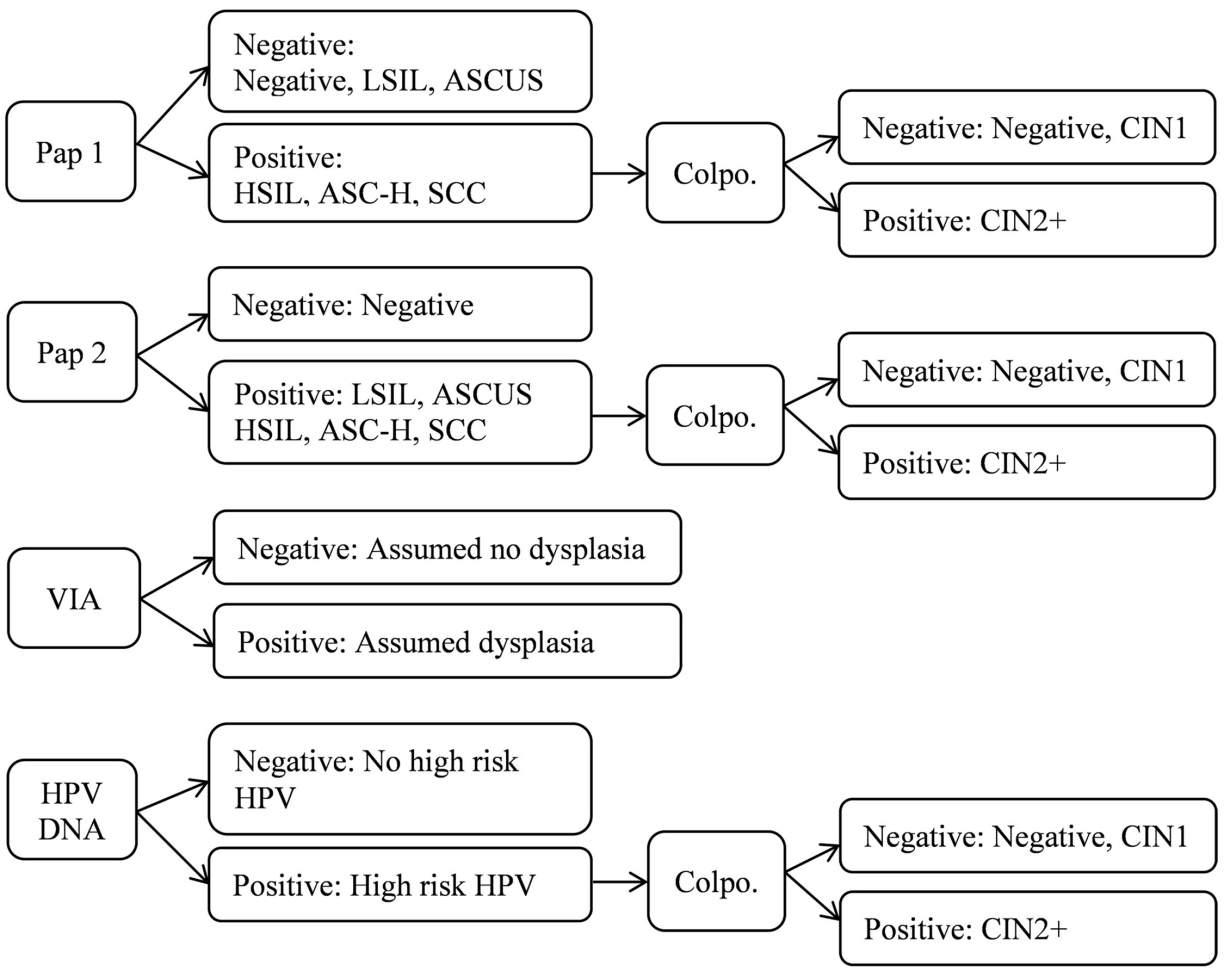
Strategies for cost-effectiveness analysis comparing cervical screening methods. Colpo. = colposcopic biopsy, LSIL = low grade squamous intraepithelial lesions, ASCUS = atypical squamous cells of undetermined significance, ASC-H = atypical squamous cells cannot rule out high grade lesion, HSIL = high grade squamous intraepithelial lesions, SCC = Squamous cell carcinoma

Cost inputs were entered into a tool designed for this analysis in Microsoft Excel [28]. Using the outcomes estimated in the validation study, the average cost per case of CIN2+ detected by each screening strategy was calculated and compared taking into consideration both the costs for the screening method alone and for colposcopic biopsy if required for women with positive screening outcomes. Although positive VIA cases were followed with colposcopic biopsy in the study for validation purposes, this would not occur in clinical practice in South Africa at present and was not included in this cost-effectiveness analysis. Fig 2 illustrates the strategies considered.

To explore the impact of scale on the cost and cost-effectiveness outcomes, two caseload scenarios were created. The Study Statistics scenario uses the average number of procedures actually done per day per nurse or doctor during the validation study. The Functional Limit scenario represents the maximum number of procedures estimated to be possible per nurse or doctor during an eight hour work day given the time required per procedure, which includes time spent with each patient as well as daily, weekly and monthly tasks required for the service. SI Tables contains further detail.

Univariate sensitivity analysis was performed to explore the impact of variation in the costing inputs, service volumes and sensitivity and specificity of each procedure on cost effectiveness. Average total costs were varied by 25% in each direction. Sensitivity and specificity were varied using the confidence interval boundaries provided in the published report from the validation study.

Finally, HPV DNA testing is not currently available in the public sector in South Africa. In consideration of the potential for its introduction, threshold analysis was used to determine the necessary reduction in costs required to make this novel approach competitive with the currently offered conventional cytology service.

Ethics approval for all data collection and analysis was obtained from the Human Ethics Research Committee at the University of the Witwatersrand. The Consolidated Health Economics Evaluation Reporting checklist, a standardized, internationally recognized tool, was used in the preparation of this manuscript [29].

## Results

### Screening services

The resources required to provide screening are summarized in Table 1. The VIA service included painting and viewing the cervix. The nurse also photographed the cervix, and in a weekly quality control meeting the photographs and diagnoses were reviewed by a team of nurses and doctors. Colposcopic biopsy was performed by a medical officer with assistance from a staff nurse. Cytology slides and histology specimens were sent to the NHLS as per standard of care for processing. Medical officers reviewed all Pap results when received from the NHLS and provided instruction on whether to call back the women for follow up. If a woman returned for her results, these were provided by a professional nurse and/or medical officer. Counsellors were available to provide extra counselling and information if needed. HPV DNA samples were sent to a special lab at the University of Cape Town, and results were emailed back to the study team.

**Table 1:** Resources utilized to provide screening services.

Resource	Details
*Personnel* *	
Staff nurse	Retrieved the day’s supplies, set up the rooms, etc. Assisted with colposcopic biopsies.
Professional nurse	Performed Pap and HPV DNA sample collection, provided screening results.
PHC nurse	Cost used for VIA screening and participation in weekly VIA quality control meeting.
Medical officer	Reviewed all Pap results, provided instruction on whether to call back the women for follow up, assisted in provision of results as needed. Performed colposcopic biopsies.
Counselor	Assisted in calling back women and scheduling return visits if needed.
*Supplies and equipment*	
Supplies	Gloves, masks, linen savers, cotton swabs, paper towels, hand washing/sanitizing supplies, pens/pencils, forms, files, sanitary towels, acetic acid, paper towels.
Furnishings	Two rooms for screening and one for colposcopic biopsy procedures contained desks, chairs, examination beds, trolleys, and other medical furnishings.
Other equipment	Speculum, metal receiving dishes, colposcope machine, punch biopsy forceps, digital camera for VIA, monitor for VIA quality control meetings, etc.
*Laboratory*	
Cytology and histology	Lab fee which included cytology collection spatula, slides, fixative, specimen vials, formalin, lab forms and materials for shipment to/from lab.
HPV DNA analysis	Hybrid capture II sample collection kit, courier fee, lab testing fee.

PHC nurse = Primary health care nurse

*Training for the nurses is as follows: Staff nurse, 2 years; Professional nurse, 4 years; PHC nurse, 6 years.

### Screening costs

The costs for screening include both service-level and per procedure inputs. Service-level inputs include equipment, certain supplies and elements of staff time. For example, the time spent each day gathering the necessary supplies, setting up and cleaning the clinic rooms, and filling out the necessary paperwork was constant regardless of the number of procedures performed. In contrast, staff time, lab fees and most supply usage varied depending on the number of procedures performed.

Table 2 provides information about the two caseload scenarios considered when estimating the cost per procedure. In the Study Statistics scenario, the same number of procedures was done per day for each screening method—roughly two procedures per day per nurse for screening and roughly three colposcopic biopsies per day per medical officer. In the Functional Limit scenario, the procedures possible per eight hour work day per nurse increased to 12.6 Paps, 11.6 VIA’s, or 14.2 HPV DNA tests. Also under this scenario, it is estimated that the medical officers could do as many as 22.7 colposcopic biopsies per day. The number of procedures theoretically possibly in this second scenario might not be practical given the interruptions expected in a typical work day; however it is helpful to consider them as an upper boundary.

**Table 2:** Caseload scenarios: Total procedures possible per day per study nurse or doctor.

Scenario	Pap	VIA	HPV	Colposcopic biopsy
	Professional nurse	PHC nurse	Professional nurse	Medical officer with assistance from staff nurse
Study Statistics*	1.9	1.9	1.9	3.3
Functional Limit**	12.6	11.6	14.2	22.7

*Average number of procedures done in the clinic during the study timeframe (i.e. average per nurse/doctor per day).

**Calculated using the reported time required per procedure plus time required for daily, weekly and monthly activities directly required by each service. It assumes 8 hours of productive time per work day.

Table 3 provides the average total cost per screening procedure given the two service volume scenarios. The base case results and a range representing 25% higher and lower costs are presented for personnel, supplies, equipment, lab and total costs. The costs were higher for all procedures under the Study Statistics scenario than under the Functional Limit scenario where volumes were higher. In both scenarios, VIA was the least costly of the three screening options at an average total cost of US$ 3.67 (2.76–4.59) under the Functional Limit scenario and US$ 9.12 (6.84–11.40) under the Study Statistics scenario. Pap testing cost US$ 8.17 (6.13–10.22) and US$ 12.55 (9.41–15.68) under the two respective scenarios; and HPV DNA testing was most expensive at US$ 54.34 (40.75–67.92) and US$ 58.61 (43.96–73.26). The difference in costs was largely explained by lab costs, which comprised the majority of the Pap and HPV DNA testing costs. VIA services do not require any external laboratory testing. HPV DNA lab costs were particularly high at US$ 51.74 (38.80–64.67) per test, or 95.2% of the total procedural cost. This high cost was likely because the test was performed by an academic lab during the study and was not yet available in the public sector. Colposcopic biopsy costs were US$ 67.71 (50.79–84.64) under the Functional Limit scenario and US$ 75.04 (56.28–93.80) under the Study Statistics scenario.

**Table 3:** Average estimated procedure costs for each scenario (USD 2013), cost ranges for sensitivity analysis.

	Functional Limit		Study Statistics**	
	Cost (Range)*	% of total	Cost (Range)*	% of total
Pap				
Personnel	1.43 (1.08–1.79)	17.6	2.64 (1.98–3.30)	21.0
Supplies	1.03 (0.77–1.29)	12.6	1.40 (1.05–1.76)	11.2
Equipment	0.50 (0.37–0.62)	6.1	3.30 (2.47–4.12)	26.3
Lab/transport	5.21 (3.91–6.51)	63.7	5.21 (3.91–6.51)	41.5
Total	8.17 (6.13–10.22)	100.0	12.55 (9.41–15.68)	100.0
VIA				
Personnel	1.56 (1.17–1.95)	42.5	2.10 (1.57–2.62)	23.0
Supplies	1.24 (0.93–1.55)	33.7	1.71 (1.28–2.14)	18.8
Equipment	0.88 (0.66–1.09)	23.8	5.32 (3.99–6.64)	58.3
Lab/transport	0.00 (0.00–0.00)	0.00	0.00 (0.00–0.00)	0.0
Total	3.67 (2.76–4.59)	100.0	9.12 (6.84–11.40)	100.0
HPV DNA				
Personnel	1.39 (1.04–1.73)	2.5	2.36 (1.77–2.95)	4.0
Supplies	0.76 (0.57–0.95)	1.4	1.14 (0.85–1.42)	1.9
Equipment	0.46 (0.34–0.57)	0.8	3.38 (2.54–4.23)	5.8
Lab/transport	51.74 (38.80–64.67)	95.2	51.74 (38.80–64.67)	88.4
Total	54.34 (40.75–67.92)	100.0	58.61 (43.96–73.26)	100.0
Colposcopic biopsy				
Personnel	2.10 (1.58–2.63)	3.1	2.26 (1.70–2.83)	3.0
Supplies	1.50 (1.12–1.87)	2.2	2.84 (2.13–3.55)	3.8
Equipment	1.00 (0.75–1.25)	1.5	6.83 (5.12–8.53)	9.1
Lab/transport	63.11 (47.33–78.89)	93.2	63.11 (47.33–78.89)	84.1
Total	67.71 (50.79–84.64)	100.0	75.04 (56.28–93.80)	100.0

*Range represents 25% lower and higher than base case. These boundaries were used for the sensitivity analysis.

**The Study Statistics estimates were also explored in the sensitivity analysis.

### Cost effectiveness

As published previously, 1,202 women underwent the three screening strategies under investigation in the validation study; 1,193 were included in the analytic cohort and cost effectiveness analysis. As indicated in Table 4, the cost of screening this population with the initial screening method only was US$ 4,383 with VIA, US$ 9,750 for Pap and US$ 64,826 with HPV DNA testing. The “true prevalence” of CIN2+ in the study population, calculated based on colposcopic biopsy results, was 32.94% (95% confidence interval 30.01–36.01) or 393 cases [13]. As indicated in Table 4, given this prevalence and the published sensitivity and specificity information, using the Pap 2 definition, cytology would have identified 888 positive cases (i.e. all abnormal results) (373 true positive, 515 false positive). HPV DNA testing resulted in detection of 750 positive cases (361 true positive, 389 false positive). The Pap 1 definition identified 431 cases (298 true positive, 133 false positive), and VIA identified 509 cases (257 true positive, 252 false positive).

**Table 4:** Screening outcomes, costs for screening analytic cohort for CIN 2+ (Functional Limit scenario).

	Pap 1		Pap 2		VIA	HPV DNA
*Sensitivity and specificity (95% CI)* [13]						
Sensitivity		75.8% (70.8–80.8)		94.8% (90.5–99.2)	65.4% (59.7–71.1)	91.9% (88.5–95.3)
Specificity		83.4% (80.9–85.9)		35.6% (32.2–38.9)	68.5% (65.3–71.7)	51.4% (48.0–54.8)
*Test results (n = 1*,*193)*						
Total positive (95% CI)		431 (430–431)		888 (879–898)	509 (506–512)	750 (736–764)
TP (95% CI)		298 (278–318)		373 (356–390)	257 (235–279)	361 (348–375)
FP (95% CI)		133 (113–153)		515 (489–542)	252 (226–278)	389 (362–416)
Missed cases (FN’s) (95% CI)		95 (75–115)		20 (3–37)	136 (114–158)	32 (18–45)
*Screening costs (US $)* *						
Initial screen		9,750 (7,313–12,188)		9,750 (7,313–12,188)	4,383 (3,287–5,478)	64,826 (48,619–81,032)
Colpo for all positive cases**		29,165 (21,891–36,427)		60,115 (45,609–74,373)	0.00 (0.00–0.00)	50,784 (38,791–62,309)
Total costs***		38,915 (29,204–48,615)		69,865 (52,922–86,561)	4,383 (3,287–5,478)	115,610 (87,410–143,341)
% of total cost spent on colpo. for FP’s		23.1%		49.9%	0.0%	22.8%
Cost per TP case detected		130.63 (104.95–153.09)		187.52 (148.79–222.02)	17.05 (14.01–19.61)	320.09 (251.31–382.71)

CI = Confidence interval, TP = True positive, FP = False positive, FN = False negative, Colpo. = colposcopic biopsy

*All costs are presented with a range of 25% higher and lower.

**Considers the colposcopic biopsy costs for true positives plus false positives. Not clinically relevant for VIA.

***For initial screen plus colposcopic biopsy when indicated. Excludes colposcopic biopsy for VIA because not clinically relevant.

For Pap 1, Pap 2 and HPV DNA screening, all positive cases incurred the additional cost of colposcopic biopsy. The total cost of screening, i.e. the cost of the initial screening method plus the colposcopic biopsy for screen positive cases, was US$ 38,915 for Pap 1, US$ 69,865 for Pap 2, and US$ 115,610 for HPV DNA testing. The total cost of screening for VIA was the cost of initial screening only—US$ 4,383. The false positives identified by Pap 1, Pap 2 and HPV DNA screening were responsible for 23.1%, 49.9% and 22.8% of the total costs respectively.

Finally, considering the cost per true positive CIN2+ case detected, VIA was least expensive with an average cost of US$ 17.05 per case. Pap 1 was US$ 130.63 per case; Pap 2 was US$ 187.52 per case; and HPV DNA screening was most costly at US$ 320.09 per case.

**Fig 3:**
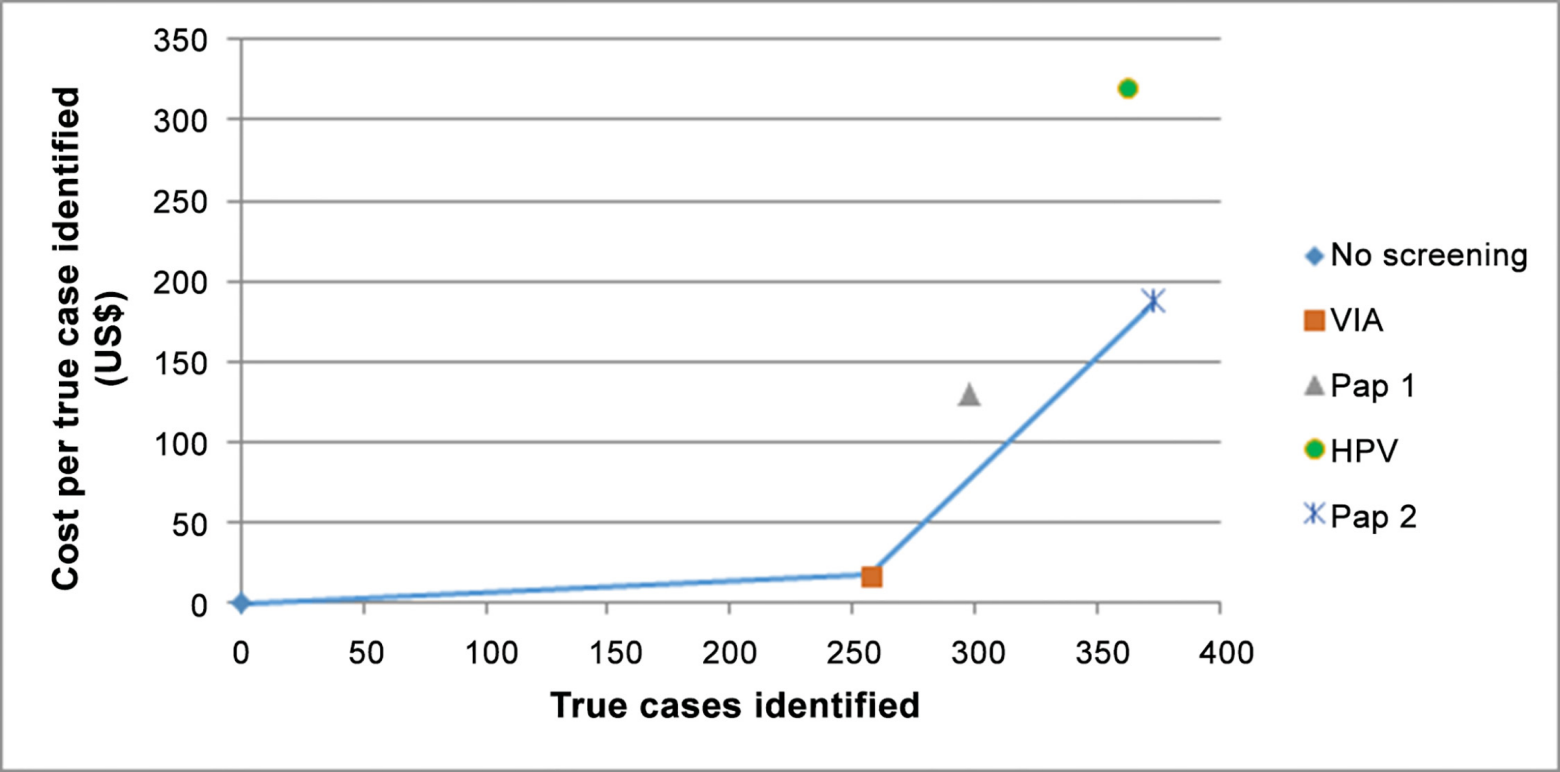
Comparison of screening methods: True cases of CIN2+ detected by cost per case. NB: The line represents the cost-effectiveness threshold, or frontier. All interventions or combinations of interventions along this line are more cost effective than intervention or combination of interventions left of the line.

Comparing the incremental cost effectiveness of the four approaches, HPV DNA screening was “dominated” by Pap 2 in that HPV DNA testing offered fewer true cases while costing more overall. Then comparing VIA, Pap 1 and Pap 2, Pap 1 was dominated by Pap 2 in that the marginal cost of identifying true CIN2+ cases was higher when compared to VIA. Table 5 provides the Incremental Cost Effectiveness Ratios (ICERs) for the final comparison. Fig 3 illustrates the cost effectiveness “frontier” when comparing the four approaches for HIV-positive women on ARV treatment. Again, VIA and Pap 2 are the dominant options.

**Table 5:** Incremental cost-effectiveness analysis (Functional Limit scenario).

	Total		Incremental		ICER
	True cases detected	Cost*	True cases detected	Cost	
No screening	0	0	—	—	—
VIA alone	257	4,383	257	4,383	17
Cytology (Pap-1) plus colpo.	298	38,915	Dominated***	Dominated***	—
HPV DNA plus colpo.	361	115,610	Dominated**	Dominated**	—
Cytology (Pap-2) plus colpo.	373	69,865	116	65,482	567

ICER = Incremental cost-effectiveness ratio, colpo. = colposcopic biopsy

*For initial screen plus colposcopic biopsy when indicated. Excludes colposcopic biopsy for VIA because not clinically relevant.

**HPV DNA screening was dominated by Pap 2 in that it offered fewer cases for a higher total cost.

***Pap 1 was dominated by Pap 2 in that the marginal cost of identifying cases was higher than with Pap 2.

In the sensitivity analysis, the comparison of total costs and ranking of methods did not change when the costs for each screening procedure varied by 25 percent higher or lower or for the different service volume scenarios. Changing the sensitivity and specificity data to the extent indicated in the 95% confidence intervals published for the validation study (and presented in Table 4) also did not change the ranking of the methods except for in one case. Increasing the sensitivity of HPV DNA screening to the higher bound of the confidence interval (i.e. to 95.3%) meant that this method was then more sensitive than Pap 2 and identified more true positive cases. However these cases still came at a higher cost of US$ 311.08 per case detected (as compared to US$ 187.52 for Pap 2).

As noted above, HPV DNA testing is not currently available in the public sector in South Africa. Reducing the lab costs required for the test to US$ 1.00 would reduce the total cost per screen to US$ 3.60 and reduce the cost per true case of CIN2+ detected to US$ 152.51. However, due to the higher sensitivity and lower specificity of HPV DNA testing as compared to Pap 1 among HIV-positive women, the cost per true case detected is still higher with HPV DNA testing than for Pap 1. In fact, due to the higher number of false positive cases identified and the cost of colposcopic biopsies for these cases, even reducing the lab costs for HPV DNA testing to zero, HPV DNA testing is still more expensive per true CIN2+ case identified than Pap 1. Pap 2 was put forward in the validation study as a hypothetical, alternative screening approach for HIV-positive women on ARV treatment. However, it is important to note that the Pap 2 option is currently not allowed within the national screening guidelines. In the incremental analysis provided in Table 5, Pap 2 offered more true cases for less cost and thus “dominated” the HPV DNA option. However, if the lab costs for HPV DNA testing were reduced by roughly 75% to US$ 12.93 (resulting in a 71% reduction of the total screening cost to US$ 15.54), the average total cost per case detected would then be US$ 191.92, and HPV DNA testing would no longer be dominated by the hypothetical Pap 2 approach (Table 6).

**Table 6:** Threshold analysis comparing the costs of HPV DNA testing to actual and hypothetical Pap screening (Functional Limit scenario).

	HPV DNA versus Pap 1		HPV DNA versus Pap 2	
	Pap 1	HPV DNA	Pap 2*	HPV DNA
Screening cost reduced to:				
Lab cost (US$)	No change	1.00	No change	12.93
Total cost (US$)	No change	3.60	No change	15.54
True cases detected	298	361	373	361
Total cost for screening**	38,915	55,083	69,865	69,320
Cost per true case detected	130.63	152.51	187.52	191.92

* Note that this method is not currently allowed under existing guidelines, but was presented as a hypothetical alternative in the validation study.

** For initial screen plus colposcopic biopsy when indicated. Excludes colposcopic biopsy for VIA because not clinically relevant.

## Discussion

Considering VIA, HPV DNA, Pap 1 and Pap 2 as four cervical cancer screening approaches for HIV-positive women on ARV treatment, VIA was most cost effective at US$ 17.05 per true case of CIN2+ detected. However, due to its lower sensitivity and specificity, using VIA resulted in a high number of missed diagnoses (i.e. false negative results). The hypothetical Pap 2 alternative (i.e. any abnormal Pap smear) had the highest sensitivity and lowest specificity of all the approaches and resulted in the highest number of true cases being detected, albeit with a large number of women having unnecessary colposcopic biopsies because all non-negative Pap results were considered positive. Colposcopic biopsy was relatively expensive, costing almost US$ 70 per procedure. When the cost of colposcopic biopsy as a follow up for screen positive cases was added for Pap 1, Pap 2 and HPV DNA screening, the total cost for screening was highest for HPV DNA screening at US$ 115,610 for the analytic cohort of 1,193 women. Colposcopic biopsy was clinically irrelevant for VIA as diagnosis and treatment could be provided the same day, so the total cost for screening was lowest with VIA and represented the initial screening cost only, i.e. US$ 4,383.

Currently in South Africa, conventional cytology using the Pap 1 definition as presented here is the screening method offered in the public sector for both HIV-negative and HIV-positive women. This analysis suggests that VIA might be a more cost-effective approach for screening HIV-positive women; however, there are several important issues for consideration. First, as noted above, VIA is less specific than the other three approaches, potentially resulting in more overtreatment. “Overtreatment” with VIA assumes that cryotherapy would be a viable treatment option and be offered the same day; whereas “overtreatment” with the other methods might involve unnecessary colposcopic biopsies. To understand the trade-off presented here, one would need to compare the risks and costs of overtreatment with cryotherapy versus the risks and costs of the unnecessary colposcopic biopsies that would follow the other screening approaches.

Second, VIA is also less sensitive for CIN2+ detection than the other three approaches, resulting in more missed cases. Clearly missing a diagnosis has serious implications, especially among HIV-positive women who are at increased risk for progression and less likely to have disease regression [9]. However, it is important to remember that the analysis presented here was conducted within a study setting where there were no barriers to access or reduced loss to follow up for any service. This is not the case in South Africa’s public sector. Similar to many other low and middle income country settings, South Africa’s public sector is plagued by high numbers of inadequate cervical smears, often as the result of poor collection techniques [30]. Women with inadequate smears must be called back for a repeat test, presenting an opportunity for loss to follow up. For women who do receive a positive, or abnormal, Pap result, many do not return for colposcopic biopsy. Data are lacking on the exact magnitude of the problem, but it is known anecdotally to be significant and in large part caused by service limitations.

Loss-to-follow-up problems are not unique to South Africa, and have been shown to be critical for bigger-picture comparisons of screening methodologies. In 2005, Goldie et al, used modelling to determine that screening strategies requiring fewer visits and less opportunity for loss to follow up were most cost-effective when considering lifetime risk of cancer, years of life saved, lifetime costs, and cost-effectiveness ratios. They determined that for five developing country locations, including South Africa, once in a lifetime screening with VIA or HPV DNA testing followed by immediate cryotherapy reduced lifetime risk of cancer by roughly 25–36% and cost less than $500 per life year saved [19].

Concerns about VIA’s sensitivity and specificity could possibly be assuaged through training and regular quality assurance activities. In Firnhaber et al’s (2013) validation study, they present sensitivity and specificity data for VIA performed by nurses as well as doctors [13]. Although the performance was comparable and global VIA recommendations do specify that the service can be safely offered by nurses [31], the sensitivity and specificity outcomes for the doctors were higher [13]. If nurses’ outcomes could be improved over time through practice and quality management efforts including a second review via digital cervicography [32], missed cases and overtreatment may be reduced. Note that this added quality assurance cost was not included in this analysis.

The hypothetical Pap 2 approach for screening, which classified any abnormal result as positive and requiring colposcopic biopsy, was also competitive in the incremental cost-effectiveness analysis. This approach casts a “wide net” and identifies almost all of the true cases in the analytic cohort; however, many women also receive false positive diagnoses. For HIV-positive women with increased risk of disease progression, perhaps the “wide net” approach is acceptable to avoid missing cases. HPV DNA testing is also highly sensitive and offers greater specificity than the Pap 2 option, but it was also very expensive under the study conditions. In the threshold analysis, with a 71% reduction in the costs of the lab component of the HPV DNA screening cost, this approach became a competitive alternative as compared to the Pap 2 approach. Economies of scale and/or new technologies may offer opportunities for lowered costs. Point-of-care (POC) technologies for HPV testing are currently being validated in South Africa and may help to eliminate expensive lab costs. Initial outlays for POC technologies are often high, and significant volumes would likely need to be guaranteed to ensure competitive pricing with this approach. However, at least one new POC option (Xpert) is currently becoming more available in areas with HIV prevalence due to its use in tuberculosis screening [33]. This machine uses self-contained testing cartridges, and the manufacturer offers a range of options, including testing for sexually transmitted infections and HPV [34].

Comparing HPV DNA testing with the currently available Pap 1 approach, it is important to consider that the total cost of screening for these two approaches (and Pap 2) are largely driven by the costs of colposcopic biopsy. Because HPV DNA testing is more sensitive and less specific than the Pap 1 approach, it results in more women undergoing colposcopic biopsy in this analysis. The extra cost for those additional women is so great that, even if HPV DNA screening costs were dropped to zero, it would remain a more costly approach in this analysis.

### Limitations

This analysis has its limitations. Considering the cost outcomes, training and overhead were not included, and retrospective collection of data within a study setting was challenging. However, the cost of the nurse for the screening procedures was varied to address the nursing education/qualification requirements of the procedures, and data were collected from at least three individuals in an attempt to reduce recall bias. Other unit costs are available in the literature but are difficult to compare given variation in the methods used to collect the costs. To our knowledge, the work presented here is the only full micro-costing of cervical cancer screening in South Africa. Others have depended all or in part on South Africa’s Uniform Patient Fee Schedule to obtain unit costs and may not accurately reflect costs to the health system [18,20,35].

Also, this analysis stops at diagnosis of CIN2+ and does not consider the cost of treating women with dysplasia or cervical cancer or the potential for cancer-related mortality. Further, the time horizon stops at identification of cases and does not consider the long term repercussions of missed cases, or false negatives. Similarly, loss to follow up between screening and colposcopic biopsy was not included as this was not a significant issue within the validation study setting. Future analyses should consider longer time horizons, the potential for loss to follow up and the cost of treatment (cryotherapy or other alternatives).

Despite these limitations, our results are similar to other published cost-effectiveness analyses from South Africa in that strategies which eliminate or reduce the need for follow up visits and/or expensive colposcopy seem most competitive. In 2001, Goldie et al considered lifetime costs and benefits, including those associated with treating cancer if found, and concluded that HPV or VIA followed by same day treatment was most cost effective [18]. In a separate modelling exercise from 2005, Goldie et al recommended HPV DNA testing again followed by same day treatment [35], and in 2009, Vijayaraghavan et al recommended conventional cytology followed by HPV DNA testing for triaging rather than colposcopic biopsy [20].

## Conclusion

Despite its limitations, this study provides locally collected cost data for three cervical dysplasia screening methods and cost effectiveness data for four screening approaches. In low-resource settings, such as South Africa, with a high burden of high-risk HPV and HIV, cost-effective strategies for cervical cancer screening are necessary if limited resources are to be used effectively. In South Africa, national strategic documents indicate an aim of 70% coverage for the current Pap services; however, actual coverage falls short. For 2010–2011, based on ten-yearly screening intervals, coverage was estimated to be 52% [36]. Locally collected cost and cost effectiveness data are important for local policymakers charged with improving these statistics.

Ultimately women need access to services which meet their needs and address the significant burden of cervical dysplasia and cancer in this region. In many resource limited settings, improving existing Pap services is a goal for addressing this need, which is particularly urgent among HIV-positive women. However, alternatives exist. VIA offers the ability for same day diagnosis and treatment, minimizing loss to follow up. Zambia’s national screening program is based on VIA and may be seen as a model for other, similar settings [37]. Alternatively, shifting to either a more conservative definition for triaging Pap results (i.e. the hypothetical Pap 2 definition) or HPV DNA testing could improve case detection, but not without increased costs. This analysis offers insight regarding the costs of three screening alternatives; however, ultimately the budget and priorities within the health system must dictate the methods selected.

## Supporting Information

S1 File Cost effectiveness analysis tables. (PDF)

## References

[1] International Agency for Research on Cancer. Cervical Cancer: Estimated incidence, mortality, and prevalence worldwide in 2012. In: GLOBOCAN 2012: Estimated Cancer Incidence, Mortality and Prevalence Worldwide [Internet]. 2012. Available: http://globocan.iarc.fr/Pages/fact_sheets_cancer.aspx

[2] World Health Organization. Global status report on noncommunicable diseases 2010. Geneva, Switzerland; 2011.

[3] Arends MJ, Buckley CH, Wells M. Aetiology, pathogenesis, and pathology of cervical neoplasia. J Clin Pathol. 1998;51: 96–103. 9602680 10.1136/jcp.51.2.96 PMC500501

[4] Bosch FX, Qiao Y-L, Castellsagué X. The epidemiology of human papillomavirus infection and its association with cervical cancer. Int J Gynecol Obstet. 2006;94: S8–S21. 10.1016/S0020-7292(07)60004-6 29644633

[5] World Health Organization. WHO Guidance Note: Comprehensive cervical cancer prevention and control: a healthier future for girls and women. Geneva, Switzerland; 2013.

[6] Franco EL, Villa LL, Sobrinho JP, Prado JM, Rousseau MC, Désy M, et al Epidemiology of acquisition and clearance of cervical human papillomavirus infection in women from a high-risk area for cervical cancer. J Infect Dis. 1999;180: 1415–23. 10.1086/315086 10515798

[7] Eckert LO, Watts DH, Koutsky L a, Hawes SE, Stevens CE, Kuypers J, et al A matched prospective study of human immunodeficiency virus serostatus, human papillomavirus DNA, and cervical lesions detected by cytology and colposcopy. Infect Dis Obstet Gynecol. 1999;7: 158–64. 10.1155/S1064744999000253 10371475 PMC1784734

[8] Duerr A, Kieke B, Warren D, Shah K, Burk R, Peipert JF, et al Human papillomavirus-associated cervical cytologic abnormalities among women with or at risk of infection with human immunodeficiency virus. Am J Obstet Gynecol. 2001;184: 584–90. 10.1067/mob.2001.111791 11262457

[9] Omar T, Schwartz S, Hanrahan CF, Modisenyane T, Tshabangu N, Golub JE, et al Progression and regression of premalignant cervical lesions in HIV-infected women from Soweto: a prospective cohort. Aids. 2011 10.1097/QAD.0b013e328340fd99 PMC3166782 21076276

[10] Firnhaber C, Westreich D, Schulze D, Williams S, Siminya M, Michelow P, et al Highly active antiretroviral therapy and cervical dysplasia in HIV-positive women in South Africa. J Int AIDS Soc. 2012;15: 17382 10.7448/IAS.15.2.17382 22713259 PMC3499783

[11] UNAIDS. South Africa: HIV and AIDS estimates [Internet]. 2012. Available: http://www.unaids.org/en/regionscountries/countries/southafrica/

[12] Allan B, Marais DJ, Hoffman M, Shapiro S, Williamson A-L. Cervical human papillomavirus (HPV) infection in South African women: implications for HPV screening and vaccine strategies. J Clin Microbiol. 2008;46: 740–2. 10.1128/JCM.01981-07 17977997 PMC2238131

[13] Firnhaber C, Mayisela N, Mao L, Williams S, Swarts A, Faesen M, et al Validation of Cervical Cancer Screening Methods in HIV Positive Women from Johannesburg South Africa. PLoS One. 2013;8: 2–9. 10.1371/journal.pone.0053494 PMC3543403 23326441

[14] Sankaranarayanan R, Gaffikin L, Jacob M, Sellors J, Robles S. A critical assessment of screening methods for cervical neoplasia. Int J Gynaecol Obstet. 2005;89 Suppl 2: S4–S12. 10.1016/j.ijgo.2005.01.009 15823266

[15] Denny L, Kuhn L, Souza M De. Screen-and-Treat Approaches for Cervical Cancer Prevention in Low-Resource Settings: A Randomized Controlled Trial. JAMA. 2005;294: 2173–2181. 10.1001/jama.294.17.2173 16264158

[16] National Department of Health Republic of South Africa. National Guidelines for Cervical Cancer Screening Programme. Pretoria, South Africa; 2002.

[17] National Department of Health South Africa. Clinical Guidelines for the management of HIV and AIDS in adults and adolescents. 2010.

[18] Goldie SJ, Kuhn L, Denny L, Pollack A, Wright TC. Policy Analysis of Cervical Cancer Screening Strategies in Low-Resource Settings: Clinical Benefits and Cost-effectiveness. JAMA. 2001;285: 3107–3116. 10.1001/jama.285.24.3107 11427139

[19] Goldie SJ, Gaffikin L, Goldhaber-Fiebert JD, Gordillo-Tobar A, Levin C, Mahé C, et al Cost-effectiveness of cervical-cancer screening in five developing countries. N Engl J Med. 2005;353: 2158–2168. 10.1056/NEJMsa044278 16291985

[20] Vijayaraghavan A, Efrusy M, Lindeque G, Dreyer G, Santas C. Cost effectiveness of high-risk HPV DNA testing for cervical cancer screening in South Africa. Gynecol Oncol. Elsevier Inc.; 2009;112: 377–383. 10.1016/j.ygyno.2008.08.030 19081611

[21] National Department of Health Republic of South Africa. Medical and Pharmaceutical Contracts [Internet]. Available: http://www.health.gov.za/mpc2.php

[22] Department of Health: Republic of South Africa. Essential Drugs Programme [Internet]. Available: http://www.health.gov.za/edp.php

[23] Department of Public Service Administration. Salary scales, with translation keys, for employees on salary levels 1 to 12 and covered by Occupation Specific Dispensations (OSDs). Pretoria; 2013.

[24] Drummond M, Sculpher M, Torrance G, O’Brien B, Stoddart G. Methods for the Economic Evaluation of Health Care Programmes. Third. Oxford: Oxford University Press; 2005.

[25] South African Revenue Service. Binding General Ruling (Income Tax): No. 7—Wear-and-tear or depreciation allowance. South Africa;

[26] Iternational Monetary Fund. Consumer Price Index. In: World Economic Outlook Database [Internet]. [cited 9 Apr 2015]. Available: https://www.imf.org/external/pubs/ft/weo/2014/02/weodata/index.aspx

[27] Historical exchange rate for 1 Jan 2013 to 31 Dec 2013 [Internet]. Available: www.oanda.com

[28] Microsoft Corporation. Microsoft Excel. Redmond, Washington;

[29] Husereau D, Drummond M, Petrou S, Carswell C, Moher D, Greenberg D, et al Consolidated Health Economic Evaluation Reporting Standards (CHEERS) statement. Value Heal. 2013;16: e1–5. 10.1016/j.jval.2013.02.010 23538200

[30] Belhadj H, Rasanathan J, Denny L, N B. Sexual and reproductive health and HIV services: integrating HIV/AIDS and cervical cancer prevention and control. Int J Gynaecol Obstet. 2013;121 Suppl: S29–34. 23477703 10.1016/j.ijgo.2013.02.002

[31] Sellors J, Lewis K, Kidula N, Muhombe K, Tsu V, Herdman C. Screening and management of precancerous lesions to prevent cervical cancer in low-resource settings. Asian Pac J Cancer Prev. 2003;4: 277–80. 14507251

[32] Firnhaber C, Mao L, Levin S, Faesen M, Examen A, Fcogsa N, et al Evaluation of a Cervicography-Based Program to Ensure Quality of Visual Inspection of the Cervix in HIV-Infected Women in Johannesburg, South Africa. J Low Genit Tract Dis. 2014;00. 10.1097/LGT.0000000000000040 PMC4272220 24914887

[33] Lawn SD, Harries AD, Meintjes G, Getahun H, Havlir D V., Wood R. Reducing deaths from tuberculosis in antiretroviral treatment programmes in sub-Saharan Africa. AIDS. 2012;26: 2121–2133. 10.1097/QAD.0b013e3283565dd1 22695302 PMC3819503

[34] Cepheid. Xpert HPV [Internet]. [cited 29 Sep 2015]. Available: http://www.cepheid.com/en/cepheid-solutions-uk/clinical-ivd-tests/sexual-health/xpert-hpv

[35] Goldie SJ, Gaffikin L, Goldhaber-Fiebert JD, Gordillo-Tobar A, Levin C, Mahé C, et al Cost-effectiveness of cervical-cancer screening in five developing countries. N Engl J Med. 2005;353: 2158–68. 10.1056/NEJMsa044278 16291985

[36] National Department of Health. Strategic Plan for Maternal, Newborn, Child and Women’ s Health (MNCWH) and Nutrition in South Africa: 2012–2016. 2012.

[37] Parham GP, Mwanahamuntu MH, Kapambwe S, Muwonge R, Bateman AC, Blevins M, et al Population-Level Scale-Up of Cervical Cancer Prevention Services in a Low-Resource Setting: Development, Implementation, and Evaluation of the Cervical Cancer Prevention Program in Zambia. PLoS One. 2015;10: e0122169 10.1371/journal.pone.0122169 25885821 PMC4401717

