# Supplementary material for: Costs and Cost Effectiveness of Three Approaches for Cervical Cancer Screening among HIV-Positive Women in Johannesburg, South Africa
Source: PLoS One. 2015 Nov 16;10(11):e0141969. doi: 10.1371/journal.pone.0141969 (PMC4646665; doi:10.1371/journal.pone.0141969)
Supplement: S1 File — (PDF) [file pone.0141969.s001.pdf]

S Table 1. Colposcopic biopsy (gold standard) results from validation study

	Colpo		
	Base	Low	High
Total women with colpo in study	941		
Total positive results	310		
"True" prevalence of CIN 2+ in study population	32.94%	30.01%	36.01%

S Table 2. Screening methods - sensitivity and specificity from validation study

							Where normal/negative = normal/LSIL/ASCUC and positive = HSIL, ASC-H, SCC			Where normal/negative = normal and positive = LSIL/ASCUS/HSIL, ASC-H, SCC		
	VIA			HPV			Pap 1			Pap 2		
Adjusted sensitivity (CIN2+)	65.4%	59.7%	71.1%	91.9%	88.5%	95.3%	75.8%	70.8%	80.8%	94.8%	90.5%	99.2%
Adjusted specificity (CIN2+)	68.5%	65.3%	71.7%	51.4%	48.0%	54.8%	83.4%	80.9%	85.9%	35.6%	32.2%	38.9%
PPV	50.5%	45.8%	55.2%	48.2%	45.5%	50.9%	69.2%	64.6%	73.8%	42.0%	39.6%	44.4%
NPV	80.1%	76.7%	83.5%	92.8%	89.5%	96.0%	87.5%	84.9%	90.1%	93.3%	87.3%	99.0%
Slide positivity rate	42.7%	42.9%	42.4%	62.9%	64.0%	61.7%	36.1%	36.1%	36.1%	74.4%	75.3%	73.7%

S Table 3. Total average costs, cost breakdowns and sensitivity analysis levers

	VIA		HPV DNA		Pap	
Personnel	1.56	0%	1.39	0%	1.43	0%
Supplies	1.24	0%	0.76	0%	1.03	0%
Equipment	0.88	0%	0.46	0%	0.50	0%
Lab	0.00	0%	51.74	0%	5.21	0%
Total procedure cost	3.67		54.34		8.17	
total comparison: sensitivity cost/cost summary	0%		0%		0%	

S Table 4. Cost effectiveness calculations

	VIA (nurse)			HPV			Pap 1			Pap 2		
	**Base**	**Low**	**High**	**Base**	**Low**	**High**	**Base**	**Low**	**High**	**Base**	**Low**	**High**
**Full cohort from validation study**												
**Total participants**	1202			1202			1202			1202		
**Inadequate sample**	1			2			6			6		
**% inadequate or inconclusive**	0.08%			0.17%			0.50%			0.50%		
**Number of women with results from screening method**	1201			1200			1196			1196		
**True positive cases**	393			393			393			393		
**True negative cases**	800			800			800			800		

Analytic cohort from validation study												
Number of women with results from all screening methods	1193			1193			1193			1193		
Total cost for screening (no colpo) (US)	4,383	3,287	5,478	64,826	48,619	81,032	9,750	7,313	12,188	9,750	7,313	12,188
Positive cases with screening method	509	512	506	750	764	736	431	431	430	888	898	879
Negative cases with screening method	684	681	687	443	429	457	762	762	763	305	295	314
True positive cases detected	257	235	279	361	348	375	298	278	318	373	356	390
Option 2 - no colpo after VIA												
Total cost for colpo for all postive screens	-	-	-	50,784	38,791	62,309	29,165	21,891	36,427	60,115	45,609	74,373
Total cost for initial screen plus colpo for positive cases	4,383	3,287	5,478	115,610	87,410	143,341	38,915	29,204	48,615	69,865	52,922	86,561
Colpo costs are x% of total	0.00%	0.00%	0.00%	43.93%	44.38%	43.47%	74.94%	74.96%	74.93%	86.04%	86.18%	85.92%
Cost per true CIN2+ case detected (w/ or without/colpo)	17.05	14.01	19.61	320.09	251.31	382.71	130.63	104.95	153.09	187.52	148.79	222.02
Wasted cost for colpo for false positives	-	-	-	26,327	21,126	30,606	8,992	7,760	9,548	34,886	27,546	41,373
Proportion of total costs spent on colpo for false positives	0.0%	0.0%	0.0%	22.8%	24.2%	21.4%	23.1%	26.6%	19.6%	49.9%	52.0%	47.8%

S Table 5a. Cost per case detected - option 2 - ICER CALCULATION STEP 1

	True cases detected	total cost	incremental cost	incremental cases	icer	Cost per case detected
No screening	0	0	0	0	0	0
VIA	257	4383	4383	257	17.05	17.05
Pap 1	298	38915	34533	41	844.85	130.63
HPV	361	115610	76695	63	1212.07	320.09
Pap 2	373	69865	-45745	11	-4013.57	187.52

S Table 5b. Cost per case detected - option 2 - ICER CALCULATION STEP 2

Cost per case detected - option 2	True cases detected	total cost	incremental cost	incremental cases	icer	Cost per case detected
No screening	0	0				0
VIA	257	4383	4383	257	17	17.05
Pap 1	298	38915	34533	41	845	130.63
HPV	361	115610	dom.	dom.		320.09
Pap 2	373	69865	30950	75	414	187.52

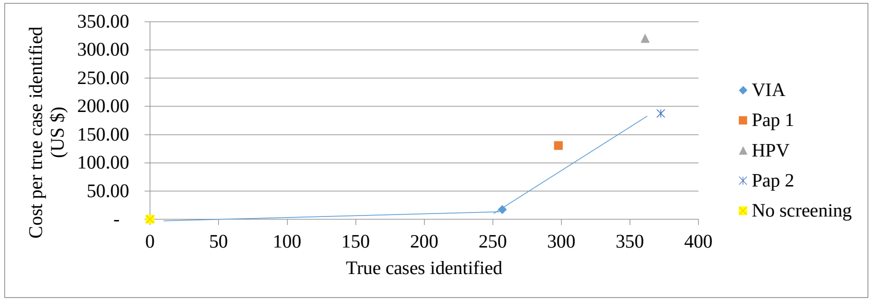

S Table 5c. Cost per case detected - option 2 - ICER CALCULATION STEP 3

**Cost per case detected - option 2**	**True cases detected**	**total cost**	incremental cost	**incremental cases**	**icer**	**Cost per case detected**
No screening	0	0	0	0	0	0
VIA	257	4383	4383	257	17	17.05
Pap 1	298	38915	34533	41	845	130.63
Pap 2	373	69865	30950	75	414	187.52

S Table 6. Cost per case detected - option 2 - VIA vs Papd 2 comparison/ranking

	**True cases detected**	**total cost**	incremental cost	**incremental cases**	**icer**	**Cost per case detected**
No screening	0	0	0	0	0	0
VIA (nurse)	257	4383	4383	257	17	17.05
Pap 2	373	69865	65482	116	567	187.52
